# Associations of Adverse Clinical Course and Ingested Substances among Patients with Deliberate Drug Poisoning: A Cohort Study from an Intensive Care Unit in Japan

**DOI:** 10.1371/journal.pone.0161996

**Published:** 2016-08-25

**Authors:** Kanako Ichikura, Yasuyuki Okumura, Takashi Takeuchi

**Affiliations:** 1 Section of Liaison Psychiatry and Palliative Medicine, Graduate School of Medical and Dental Sciences, Tokyo Medical and Dental University, Tokyo, Japan; 2 Research Department, Institute for Health Economics and Policy, Association for Health Economics Research and Social Insurance and Welfare, Tokyo, Japan; 3 Section of Psychiatry and Behavioral Sciences, Graduate School of Medical and Dental Sciences, Tokyo Medical and Dental University, Tokyo, Japan; Azienda Ospedaliero Universitaria Careggi, ITALY

## Abstract

**Objectives:**

Some patients with deliberate drug poisoning subsequently have an adverse clinical course. The present study aimed to examine whether the type of drugs ingested and psychiatric diagnoses were related to an adverse clinical course.

**Methods:**

We conducted a cohort study of patients with deliberate drug poisoning admitted to the intensive care unit of a university hospital located in Tokyo, Japan, between September 2006 and June 2013. Intensive care unit (ICU) stay of ≥4 days was used as a primary outcome measure, while the incidence of aspiration pneumonitis was used as a secondary outcome measure. Ingested substances and psychiatric diagnoses were used as explanatory variables.

**Results:**

Of the 676 patients with deliberate drug poisoning, 88% had a history of psychiatric treatment and 82% had ingested psychotropic drugs. Chlorpromazine-promethazine-phenobarbital combination drug (Vegetamin^®^) ranked fifth among the most frequently ingested substances in cases of deliberate drug poisoning and had the highest incidence of prolonged ICU stay (20%) and aspiration pneumonitis (29%). The top three major classes consisted of benzodiazepines (79%), new-generation antidepressants (25%), and barbiturates/non-barbiturates (23%). Barbiturate overdose was independently associated with increased odds of both prolonged ICU stay (8% vs. 17%; odds ratio [OR], 2.97; 95% confidence interval [CI], 1.60–5.55) and aspiration pneumonitis (8% vs. 24%; OR, 3.83; 95% CI, 2.18–6.79) relative to those associated with overdose of only other sedative-hypnotics (i.e., benzodiazepines).

**Conclusion:**

These results suggest that judicious prescribing of barbiturates by psychiatrists could reduce the risk of an adverse clinical course when a patient attempts an overdose.

## Introduction

Drug poisoning is a worldwide public health concern and places a serious burden on emergency medical service. In Western countries, the annual incidence of drug poisoning has been estimated at 142–232 per 100,000 inhabitants [1–3] and drug poisoning accounts for 0.3%–0.6% of all patients admitted to emergency departments [4, 5]. Deliberate drug poisoning is responsible for 60%–78% of all drug poisoning and is a more common cause of admission than accidental drug poisoning [6, 7].

A non-negligible subgroup of patients with deliberate drug poisoning may show an adverse clinical course such as aspiration pneumonia [8, 9], respiratory failure [10], hypothermia [9], and cardiovascular events [11], although almost all (99%) patients survive their hospital stay [12–14]. Aspiration pneumonia is the most common adverse event among patients with deliberate drug poisoning [8] and doubles the length of intensive care unit (ICU) stay owing to physical recovery from the overdose-related complications [10]. Indeed, 27%–28% of patients develop aspiration pneumonia [10, 15]. In addition, 35% of patients are hospitalized for more than 3 days in acute care hospitals [16], although these patients are generally discharged from the ICU within only 16–32 hours of admission [4, 10].

Several factors contribute to an adverse clinical course among patients with drug poisoning. For example, demographic characteristics (i.e., sex [17, 18] and age [18]), clinical characteristics (i.e., blood pressure [10, 19, 20], renal function [10], and level of consciousness [15, 19, 21]), and characteristics of the drugs ingested (dosage [20] and type [15]) are important contributing factors in adverse clinical courses among patients with drug poisoning. The dose and type of ingested drugs are particularly preventable factors associated with a severe adverse clinical course among patients with deliberate drug poisoning [22].

Although few studies have explored the associations between adverse clinical courses and ingested drugs in patients with deliberate drug overdose [15, 23–26], some specific drugs might increase the risk of an adverse clinical course. Tricyclic antidepressant poisoning leads to arrhythmia and an increased rate of mortality [23]. Barbiturate poisoning leads to respiratory depression and an increased rate of mortality [15, 24, 25]. However, these studies had several limitations. First, the specific types of drugs ingested were the focus in each of these studies and outcomes were not compared with those of other drugs. Second, some patients in these studies attempted both self-poisoning and self-cutting, which might have confounded the relationship between the type of drugs ingested and the subsequent adverse clinical course. Finally, participants were limited to patients referred to psychiatry departments [15, 23], indicating a probable selection bias. Few studies are available on the relationship between psychiatric diagnoses and adverse clinical courses, although psychiatric diagnoses may be related to the type and dose of the drugs ingested.

Therefore, in this study, we aimed to compare the incidences of aspiration pneumonia and prolonged ICU stay by the class of drugs ingested and the psychiatric diagnoses of the patients.

## Materials and Methods

### Design and setting

We conducted a cohort study of inpatients who presented with deliberate self-harm. Patients were consecutively recruited from the ICU of the Tokyo Medical and Dental University in Tokyo, Japan. The unit provides tertiary emergency service that treats 10,000 to 15,000 patients annually. Since 2006, both physicians and psychiatrists in the unit have routinely assessed clinical information for all patients who survived self-harm episodes by using a standard data extraction form. In the present study, we included patients who (1) were admitted to the unit between September 11, 2006 and June 21, 2013; (2) attempted overdose as a single method for deliberate self-harm; and (3) had complete information on the ingested substances. The present study was reviewed and approved by the institutional review board at the Tokyo Medical and Dental University (1604). Because patient data were routinely collected, the review board waived the requirement for informed consent.

### Outcomes

The incidence of prolonged ICU stay (≥4 days) was used as the primary outcome. We selected this threshold value because 90% of patients were discharged from the ICU within 3 days in our data. In addition, patients are more likely to be discharged from the ICU within 3 days rather than within 4 days because the per-day fee for admission among patients with an ICU stay of 4–7 days is 10% lower than that for a stay of 1–3 days. Indeed, the difference in the cumulative proportion of ICU stay peaked at 44% between day 1 (30%) to day 2 (77%), followed by 13% between day 2 (77%) and day 3 (90%), dropped to 4% between day 3 (90%) and day 4 (94%), and remained at 3% between day 4 (94%) and day 5 (97%).

The incidence of aspiration pneumonitis during the ICU stay was used as the secondary outcome. Patients presenting with clinical signs of pneumonia (e.g., fever ≥37.5°C, leukocytosis, and elevated C-reactive protein level) routinely undergo chest radiography and, if necessary, undergo chest computed tomography scan. Physicians in the ICU generally suspect the presence of aspiration pneumonitis on the basis of an episode of aspiration, the presence of predisposing conditions (e.g., neurological disorders, coma, and gastroesophageal reflux disease), chest radiography findings, and results of sputum culture. In this study, no distinction was made between aspiration pneumonitis and aspiration pneumonia [27].

### Primary explanatory variables

Substances that led to ICU admissions and psychiatric diagnoses were used as primary explanatory variables. Each substance was assessed by the physicians based on information from the ambulance crews. Psychiatric diagnoses were assessed according to the Diagnostic and Statistical Manual of Mental Disorders, 4th Edition (DSM-IV) criteria by experienced psychiatrists.

### Data analysis

First, we conducted univariate analyses to summarize the characteristics of the participants. Next, we selected the 20 most frequently ingested substances and major drug classes that lead to ICU admissions for overdose. Third, we identified major psychiatric diagnoses among patients with deliberate drug poisoning. Fourth, we estimated the odds ratios (ORs) and their 95% confidence intervals (CIs) for prolonged ICU stay and aspiration pneumonitis using logistic regression models. We sequentially introduced groups of variables into the model: first each primary explanatory variable, second age and sex, third psychiatric diagnoses, fourth drug classes ingested, and fifth alcohol intake during overdose. The primary explanatory variables were major drug classes and psychiatric diagnoses. The reference categories for sedative-hypnotics, antidepressants, and antipsychotics were as follows: only benzodiazepine receptor agonists or other sedative-hypnotics, only new-generation antidepressants or other antidepressants, and only second-generation antipsychotics, respectively. The reference group for mutually exclusive psychiatric diagnoses (adjustment disorders, bipolar disorders, major depressive disorders, and schizophrenia) was adjustment disorders. Finally, we performed a sensitivity analysis in which we used the prolonged length of stay as count data (i.e., days in the ICU) instead of binary data (i.e., ≥4 days or <4 days in the ICU). We estimated the incidence rate ratios (IRRs) and their 95% CIs for prolonged ICU stay using a quasi-Poisson model [28]. Significance levels were set at 5%. All data were analyzed using R version 3.2.2.

## Results

### Characteristics of the study participants

**Fig 1:**
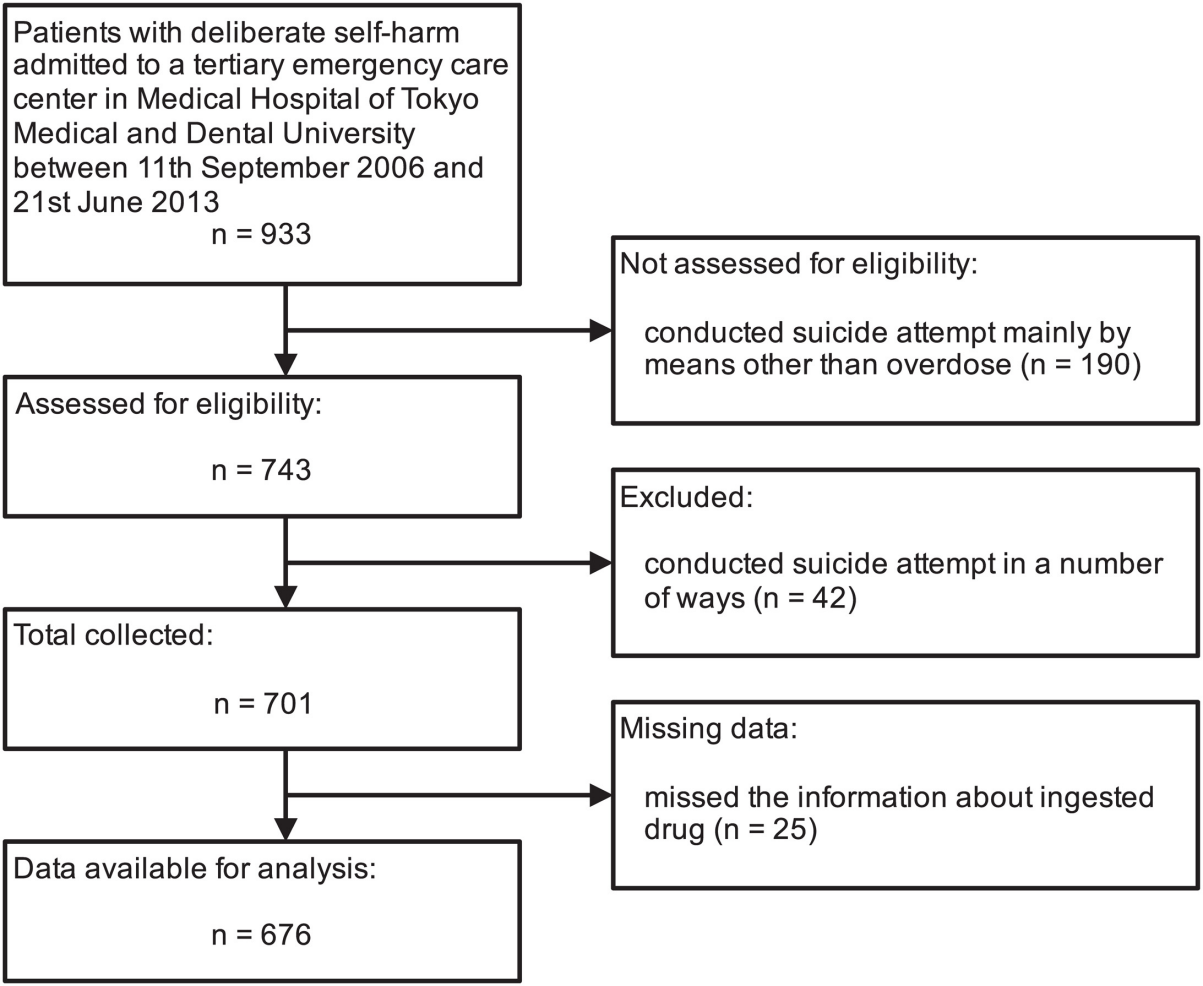
Flow diagram for inclusion in the study.

Of all the 933 patients who presented with self-harm, 676 patients that presented only with overdose were included in the analysis (see Fig 1). Characteristics of the study participants are listed in Table 1. Over 80% of patients with overdose were 20–49 years of age. The gender ratio (women: men) was 3.3:1. Among patients with overdose, 86% had a history of psychiatric treatment, 88% impulsively attempted overdose, and 60% attempted overdose with suicidal ideation.

**Table 1:** Characteristics of the study participants.

	Total (N = 676)	
Characteristic	n	%
Age		
≤19 years	38	5.6
20–34 years	328	48.5
35–49 years	227	33.6
50–64 years	47	6.9
≥65 years	36	5.3
Gender		
Men	156	23.1
Women	520	76.9
Living arrangement		
Alone	179	26.5
With family	494	73.1
Unknown	3	0.4
History of psychiatric treatment	581	87.6
Reason for self-harm		
Impulsive	592	87.6
Planned	52	7.7
Hallucination/delusion	15	2.2
Others	7	1.0
Unknown	10	1.5
Suicidal ideation		
With	403	59.9
Without	188	27.9
Unknown	82	12.2
Drugs ingested		
Psychotropic drug	557	82.4
Over-the-counter drug	103	15.2
Other drugs	16	2.4
Alcohol intake with overdose	468	69.2

### Major substance and drug classes for overdose

The top 20 substances that led to an overdose-related admission to the ICU are listed in Table 2. The top four substances were flunitrazepam, etizolam, brotizolam, and zolpidem, which are classified as benzodiazepine receptor agonists. Chlorpromazine-promethazine-phenobarbital combination drug (Vegetamin^®^) ranked as the fifth most common substance and had the highest incidence of prolonged ICU stay (20%) and aspiration pneumonitis (29%).

**Table 2:** The top 20 substances that led to ICU admission for overdose.

	Total (n = 676)		Incidence (%)^a^	
Substance	n	%	Prolonged ICU stay	Aspiration pneumonitis
Total	676	100.0	10.2	10.7
1. Flunitrazepam	178	26.3	10.1	15.7
2. Etizolam	121	17.9	8.3	10.7
3. Brotizolam	113	16.7	9.7	12.4
4. Zolpidem	105	15.5	8.6	11.4
5. Chlorpromazine-promethazine-phenobarbital	104	15.4	20.2	28.8
6. Triazolam	103	15.2	10.7	13.6
7. Bromazepam	91	13.5	12.1	16.5
8. Alprazolam	89	13.2	7.9	10.1
9. Valproate	82	12.1	7.3	8.5
10. Nitrazepam	71	10.5	9.9	16.9
11. Levomepromazine	63	9.3	4.8	15.9
12. Acetaminophen	61	9.0	11.5	0.0
13. Paroxetine	59	8.7	13.6	15.3
14. Fluvoxamine	58	8.6	12.1	17.2
15. Lorazepam	48	7.1	8.3	16.7
16. Risperidone	48	7.1	4.2	4.2
17. Diazepam	43	6.4	7.0	11.6
18. Estazolam	43	6.4	14.0	18.6
19. Chlorpromazine	41	6.1	14.6	17.1
20. Clonazepam	41	6.1	7.3	7.3

^a^ The denominator was the number of patients who ingested a substance and the numerator was the number of incident cases among the patients.

ICU = intensive care unit

The major drug classes that led to ICU admission for overdose are listed in Table 3. The top three major classes consisted of benzodiazepines (79%), new-generation antidepressants (25%), and barbiturates/non-barbiturates (23%). Among these major drug classes, barbiturates/non-barbiturates had the highest incidence of prolonged ICU stay (17%) and aspiration pneumonitis (24%).

**Table 3:** Major classes of overdose.

	Total (n = 676)		Incidence (%)^a^	
Class	n	%	Prolonged ICU stay	Aspiration pneumonitis
Total	676		10.2	10.7
1. Benzodiazepine receptor agonists	537	79.4	9.7	11.5
2. New-generation antidepressants	169	25.0	9.5	11.8
3. Barbiturates/non-barbiturates	155	22.9	17.4	23.9
4. First-generation antipsychotics	148	21.9	10.1	13.5
5. Pain killers	135	20.0	7.4	3.7
6. Second-generation antipsychotics	131	19.4	9.2	13.0
7. Mood stabilizers	117	17.3	7.7	9.4
8. Other classes	116	17.2	5.2	3.4
9. Drugs affecting the gut	77	11.4	7.8	11.7
10. Tricyclic antidepressants	72	10.7	9.7	18.1
11. Antiparkinson drugs	71	10.5	14.1	22.5
12. Other antidepressants	63	9.3	12.7	11.1
13. Antiallergic drugs	31	4.6	16.1	3.2
14. Other sedatives and hypnotics	32	4.7	15.6	6.3
15. Cardiovascular drugs	19	2.8	15.8	5.3
16. Anticonvulsants	4	0.6	25.0	0.0

^a^ The denominator was the number of patients who ingested a substance and the numerator was the number of incident cases among the patients.

ICU = intensive care unit

### Major psychiatric diagnoses

The major psychiatric diagnoses are listed in Table 4. The top three major diagnoses were major depressive disorders (26%), adjustment disorders (23%), and borderline personality disorder (21%). Amongst the major psychiatric diagnoses, borderline personality disorder had the highest incidence of prolonged ICU stay (28%) while alcohol use disorders had highest incidence of aspiration pneumonitis (31%).

**Table 4:** Psychiatric diagnoses.

	Total (n = 676)		Incidence (%)^a^	
Diagnosis	n	%	Prolonged ICU stay	Aspiration pneumonitis
Total	676		10.2	10.7
1. Major depressive disorders	174	25.7	13.8	10.3
2. Adjustment disorders	157	23.2	7.0	7.0
3. Borderline personality disorders	148	21.9	8.8	12.2
4. Schizophrenia	72	10.7	16.7	18.1
5. Bipolar disorders	59	8.7	6.8	13.6
6. Anxiety disorders	56	8.3	5.4	10.7
7. Other personality disorders	29	4.3	27.6	6.9
8. No psychiatric diagnoses	17	2.5	11.8	0.0
9. Substance use disorders	15	2.2	13.3	13.3
10. Alcohol use disorders	13	1.9	15.4	30.8
11. Eating disorders	9	1.3	0.0	11.1
12. Dementia	6	0.9	16.7	16.7

^a^ The denominator was the number of patients who have a psychiatric diagnosis and the numerator was the number of incident cases among the patients.

ICU = intensive care unit

### Risk factors for adverse clinical courses

The adjusted ORs for prolonged ICU stay and aspiration pneumonitis are shown in Table 5. Barbiturate/non-barbiturate overdose was independently associated with increased odds for a prolonged ICU stay (OR: 2.97; 95% CI: 1.60, 5.55) and aspiration pneumonitis (OR: 3.83; 95% CI: 2.18, 6.79) relative to overdose with only other sedative-hypnotics (i.e., benzodiazepine overdose). These associations were not attenuated after adjustment for other risk factors (S1 and S2 Tables).

**Table 5:** Risk factors for adverse clinical course.

		Incidence (%)^a^		Odds ratio (95% confidence interval)^b^	
Characteristic	n	Prolonged ICU stay	Aspiration pneumonitis	Prolonged ICU stay	Aspiration pneumonitis
Sedative-hypnotics					
Only benzodiazepine receptor agonists/others	426	8.2	7.7	ref	ref
Barbiturates/non-barbiturates	155	17.4	23.9	2.97 (1.60, 5.55)*	3.83 (2.18, 6.79)*
No sedative-hypnotics	95	7.4	2.1	1.03 (0.36, 2.62)	0.35 (0.05, 1.30)
Antidepressants					
Only new-generation antidepressants/others	168	8.3	10.7	ref	ref
Tricyclic antidepressants	72	9.7	18.1	0.85 (0.27, 2.42)	2.25 (0.93, 5.38)
No antidepressants	436	11.0	9.4	1.18 (0.58, 2.53)	0.85 (0.44, 1.71)
Antipsychotics					
Only second-generation antipsychotics	92	9.8	12.0	ref	ref
First-generation antipsychotics	148	10.1	13.5	0.78 (0.30, 2.08)	1.02 (0.43, 2.53)
No antipsychotics	436	10.3	9.4	0.98 (0.43, 2.46)	1.12 (0.50, 2.66)
Other classes (ref = without each drug class)					
Mood stabilizers	117	7.7	9.4	0.75 (0.30, 1.69)	0.56 (0.24, 1.21)
Antiparkinson drugs	71	14.1	22.5	1.76 (0.70, 4.17)	2.77 (1.26, 5.96)*
Pain killers	135	7.4	3.7	1.27 (0.54, 2.83)	0.59 (0.19, 1.53)
Antiallergy drugs	31	16.1	3.2	2.67 (0.78, 7.85)	0.28 (0.01, 1.61)
Cardiovascular drugs	19	15.8	5.3	0.97 (0.19, 3.74)	0.14 (0.01, 0.91)*
Drugs affecting the gut	77	7.8	11.7	0.59 (0.20, 1.49)	1.10 (0.44, 2.53)
Other	120	5.8	3.3	0.59 (0.22, 1.34)	0.23 (0.06, 0.64)*
Major diagnosis					
Adjustment disorders	156	7.1	7.1	ref	ref
Bipolar disorders	59	6.8	13.6	1.37 (0.33, 4.83)	2.07 (0.65, 6.39)
Major depressive disorders	174	13.8	10.3	1.82 (0.77, 4.51)	0.87 (0.36, 2.18)
Schizophrenia	72	16.7	18.1	2.65 (0.95, 7.54)	1.81 (0.68, 4.86)
Other	215	8.4	10.2	0.73 (0.27, 1.99)	0.70 (0.25, 1.96)
Other diagnoses					
Borderline personality disorders	148	8.8	12.2	2.87 (1.13, 7.14)*	1.86 (0.79, 4.27)
Other personality disorders	29	27.6	6.9	6.50 (2.15, 19.12)*	0.90 (0.13, 3.79)
Anxiety disorders	56	5.4	10.7	1.18 (0.25, 4.21)	1.54 (0.46, 4.65)
Substance use disorders	15	13.3	13.3	1.63 (0.21, 7.91)	0.85 (0.11, 4.26)
Alcohol use disorders	13	15.4	30.8	1.49 (0.17, 8.07)	5.40 (1.13, 23.01)*

^a^ The denominator was the number of patients who ingested a substance or who have a psychiatric diagnosis and the numerator was the number of incident cases among the patients.

^b^ Adjusted for sex, age, psychiatric diagnoses, drug classes ingested, and alcohol intake.

ICU = intensive care unit; ref = reference group.

* *p* < 0.05.

The incidence of prolonged ICU stay was 27.6% (8 of 29) in patients with other personality disorders and 9.4% (61 of 647) in those without (OR: 6.50; 95% CI: 2.15, 19.12). In addition, the incidence of prolonged ICU stay was 8.8% (13 of 148) in patients with borderline personality disorder and 10.6% (56 of 528) in those without (OR: 2.87; 95% CI: 1.13, 7.14). In the analysis of aspiration pneumonitis, there were no statistically significant differences between patients with and without personality disorders. The proportion of inter-hospital transfers was similar among patients with and without borderline personality disorder (4.1% vs. 4.7%) and was higher among patients with other personality disorders than among those without (10.3% vs. 4.3%).

The association between barbiturate overdose and prolonged length of stay was robust in the sensitivity analysis (S3 Table). Although the directions of the associations between personality disorders and prolonged length of stay were maintained, the associations did not reach statistical significance in the sensitivity analysis (S3 Table).

## Discussion

Our study yielded three major findings. First, we observed that an overwhelming majority (79%) of the patients ingested benzodiazepines that were four of the top 5 substances involved in an overdose-related admission to the ICU. Our findings differ from those of previous studies conducted in other countries [2, 7]. For example, paracetamol and ibuprofen were the two most frequently ingested substances involved in overdose-related visits to an emergency department in a teaching hospital in the United Kingdom [7]. In addition, opiates and benzodiazepines were the top 2 major drug classes involved in overdose-related visits to emergency departments in the United States [2]. One potential explanation for this discrepancy is that the availability of drugs and prescribing practice may vary from country to country.

Second, we observed that barbiturate overdose increased the odds of aspiration pneumonitis compared to other sedative-hypnotic overdose (i.e., benzodiazepine overdose). One potential explanation for the adverse effects is that barbiturates have respiratory depressant effects [15, 24]. To our knowledge, this study is the first to quantify the comparative risk of aspiration pneumonitis between a barbiturate and benzodiazepine overdose, even though the results are similar to those of previous studies that showed the comparative risk between poisoning by barbiturates and that by any other drug [15, 29]. In addition, a previous study showed that ingestion of opioids increased the risk of aspiration pneumonitis compared to non-ingestion [21]; however, this association was not replicated in our study. One potential explanation for this discrepancy is that opioids are much less consumed in Japan than in other countries [30]. Furthermore, a previous study showed that ingestion of tricyclic antidepressants increased the risk of aspiration pneumonitis compared to non-ingestion [18]. Our results did not confirm this significant association between tricyclic antidepressants and aspiration pneumonitis, although the association remained in the same direction.

Our study also adds to the literature demonstrating that barbiturate overdose increased the odds of prolonged ICU stay. Patients with barbiturate poisoning sometimes require treatment for aspiration pneumonitis and consequently have an increased risk of prolonged ICU stay. In addition, our study showed that 23% of patients presented poisoning by barbiturates. The non-negligible share of poisoning by barbiturates among overdose patients can be explained by the fact that 3% of psychiatric outpatients still receive a prescription for barbiturates in Japan [31]. Since barbiturates have been replaced by benzodiazepines as the most commonly prescribed sedatives, there has been a marked reduction in overdose-related deaths by barbiturates in the United Kingdom and India [32]. These results suggest that judicious prescription of barbiturates by psychiatrists could reduce the risk of an adverse clinical course when a patient attempts overdose.

Third, our study shows that personality disorders are associated with increased odds of prolonged ICU stay but not aspiration pneumonitis. Similar results for aspiration pneumonitis have been reported by a previous study that demonstrated no significant differences in overdose-related treatments between personality disorders and mood disorders [33]. However, our results extend this by showing that personality disorders are associated with an increased risk of prolonged ICU stay. One potential explanation for the relationship between other personality disorders and prolonged ICU stay is the coordination of inter-hospital transfer, although the mechanisms through which borderline personality contributed to a prolonged ICU stay remains unknown. This hypothesis is partly supported by the fact that patients with other personality disorders represent a higher proportion of those who require inter-hospital transfer. These patients are likely to demonstrate a greater severity of mental disorders that require acute intensive psychiatric care in a crisis stabilization unit.

Our study has three major limitations. First, the accuracy of information on the ingested drugs and psychiatric diagnoses was unknown. The data on the ingested drugs were based on reports by the ambulance crew. Psychiatric diagnoses were made only during the ICU stay. Second, a selection bias might be present because this was a single-center study. Our study population was recruited from a tertiary emergency medical facility in Japan and may have a higher frequency of patients with a severe clinical course. Third, this study lacks information on clinical, physiological, and procedural characteristics (e.g., coma at admission, acute severity scores, duration of mechanical ventilation) that might affect the risk of an adverse clinical course. Therefore, we cannot identify whether aspiration pneumonitis is associated with level of consciousness alteration or with a specific effect of the barbiturate used.

## Conclusions

Our cohort study shows increased odds for adverse clinical courses in cases of barbiturate overdose. Our results suggest that judicious prescription of barbiturates by psychiatrists could reduce the risk of an adverse clinical course when a patient attempts overdose.

## Supporting Information

S1 Table Risk factors for prolonged length of stay using logistic regression models. (PDF)

S2 Table Risk factors for aspiration pneumonitis using logistic regression models. (PDF)

S3 Table Risk factors for prolonged length of stay using a quasi-Poisson model. (PDF)
