# Supplementary material for: Associations of Adverse Clinical Course and Ingested Substances among Patients with Deliberate Drug Poisoning: A Cohort Study from an Intensive Care Unit in Japan
Source: PLoS One. 2016 Aug 25;11(8):e0161996. doi: 10.1371/journal.pone.0161996 (PMC4999209; doi:10.1371/journal.pone.0161996)
Supplement: S2 Table — (PDF) [file pone.0161996.s002.pdf]

S2 Table. Risk factors for aspiration pneumonitis using logistic regression models.

Characteristic	Odds ratio (95% confidence interval)				
	Model 1[a]	Model 2[b]	Model 3[c]	Model 4[d]	Model 5[e]
Sedative-hypnotics (ref = only benzodiazepine receptor agonists/others)					
Barbiturates/non-barbiturates	3.73 (2.24, 6.26)*	3.86 (2.30, 6.52)*	3.80 (2.21, 6.57)*	3.86 (2.20, 6.84)*	3.83 (2.18, 6.79)*
No sedative-hypnotics	0.26 (0.04, 0.86)*	0.26 (0.04, 0.89)*	0.25 (0.04, 0.86)*	0.36 (0.05, 1.34)	0.35 (0.05, 1.30)
Antidepressants (ref = only new-generation antidepressants/others)					
Tricyclic antidepressants	1.84 (0.83, 3.96)	1.74 (0.78, 3.79)	1.89 (0.83, 4.21)	2.25 (0.93, 5.37)	2.25 (0.93, 5.38)
No antidepressants	0.86 (0.49, 1.59)	0.82 (0.46, 1.50)	0.81 (0.45, 1.53)	0.85 (0.44, 1.71)	0.85 (0.44, 1.71)
Antipsychotics (ref = only second-generation antipsychotics)					
First-generation antipsychotics	1.15 (0.53, 2.60)	1.10 (0.51, 2.50)	1.08 (0.49, 2.49)	1.02 (0.43, 2.52)	1.02 (0.43, 2.53)
No antipsychotics	0.76 (0.39, 1.62)	0.73 (0.37, 1.55)	0.92 (0.45, 2.01)	1.10 (0.49, 2.61)	1.12 (0.50, 2.66)
Other classes (ref = without each drug class)					
Mood stabilizers	0.85 (0.41, 1.60)	0.89 (0.43, 1.70)	0.72 (0.33, 1.43)	0.57 (0.24, 1.23)	0.56 (0.24, 1.21)
Antiparkinson drugs	2.85 (1.50, 5.21)*	2.88 (1.50, 5.29)*	2.30 (1.16, 4.36)*	2.74 (1.25, 5.90)*	2.77 (1.26, 5.96)*
Pain killers	0.27 (0.09, 0.63)*	0.28 (0.10, 0.66)*	0.30 (0.10, 0.71)*	0.59 (0.19, 1.52)	0.59 (0.19, 1.53)
Antiallergy drugs	0.27 (0.02, 1.29)	0.28 (0.02, 1.38)	0.30 (0.02, 1.48)	0.28 (0.01, 1.64)	0.28 (0.01, 1.61)
Cardiovascular drugs	0.46 (0.03, 2.27)	0.42 (0.02, 2.09)	0.31 (0.02, 1.67)	0.14 (0.01, 0.95)*	0.14 (0.01, 0.91)*
Drugs affecting the gut	1.13 (0.50, 2.26)	1.15 (0.51, 2.33)	1.02 (0.45, 2.10)	1.13 (0.45, 2.60)	1.10 (0.44, 2.53)
Other	0.25 (0.07, 0.61)*	0.25 (0.08, 0.63)*	0.24 (0.07, 0.62)*	0.23 (0.06, 0.64)*	0.23 (0.06, 0.64)*
Major diagnosis (ref = adjustment disorders)					
Bipolar disorders	2.07 (0.76, 5.40)	2.05 (0.75, 5.44)	1.98 (0.72, 5.30)	2.13 (0.67, 6.54)	2.07 (0.65, 6.39)
Major depressive disorders	1.52 (0.70, 3.43)	1.39 (0.63, 3.20)	1.29 (0.57, 3.00)	0.89 (0.37, 2.22)	0.87 (0.36, 2.18)
Schizophrenia	2.90 (1.23, 6.97)*	2.79 (1.16, 6.80)*	2.89 (1.20, 7.10)*	1.87 (0.71, 5.00)	1.81 (0.68, 4.86)
Other	1.50 (0.72, 3.31)	1.56 (0.74, 3.47)	0.91 (0.36, 2.39)	0.70 (0.26, 1.98)	0.70 (0.25, 1.96)
Other diagnoses (ref = without each diagnosis)					
Borderline personality disorders	1.22 (0.67, 2.11)	1.46 (0.78, 2.67)	1.98 (0.91, 4.19)	1.83 (0.78, 4.19)	1.86 (0.79, 4.27)
Other personality disorders	0.61 (0.10, 2.10)	0.54 (0.08, 1.89)	0.86 (0.13, 3.27)	0.93 (0.13, 3.91)	0.90 (0.13, 3.79)
Anxiety disorders	1.01 (0.38, 2.27)	1.14 (0.42, 2.61)	1.75 (0.57, 4.81)	1.51 (0.45, 4.55)	1.54 (0.46, 4.65)
Substance use disorders	1.30 (0.20, 4.83)	1.39 (0.21, 5.22)	1.66 (0.24, 7.22)	0.89 (0.12, 4.46)	0.85 (0.11, 4.26)
Alcohol use disorders	3.89 (1.03, 12.29)*	3.53 (0.93, 11.31)	4.38 (1.09, 15.16)*	4.92 (1.05, 20.38)*	5.40 (1.13, 23.01)*

[a] Unadjusted.
[b] Adjusted for sex and age.
[c] Adjusted for sex, age, and psychiatric diagnoses.
[d] Adjusted for sex, age, psychiatric diagnoses, and drug classes ingested.
[e] Adjusted for sex, age, psychiatric diagnoses, drug classes ingested, and alcohol intake.
ref = reference group.
* $p < 0.05$.
