# Supplementary material for: Associations of Adverse Clinical Course and Ingested Substances among Patients with Deliberate Drug Poisoning: A Cohort Study from an Intensive Care Unit in Japan
Source: PLoS One. 2016 Aug 25;11(8):e0161996. doi: 10.1371/journal.pone.0161996 (PMC4999209; doi:10.1371/journal.pone.0161996)
Supplement: S3 Table — (PDF) [file pone.0161996.s003.pdf]

S3 Table. Risk factors for prolonged length of stay using a quasi-Poisson model.

Characteristic	n	M	SD	Incidence rate ratio (95% confidence interval)
Total	676	2.3	2.4	
Sedative-hypnotics				
Only benzodiazepine receptor agonists/others	426	2.2	2.2	ref
Barbiturates/non-barbiturates	155	2.7	3.0	1.21 (1.02, 1.43)*
No sedative-hypnotics	95	2.2	2.0	0.95 (0.75, 1.19)
Antidepressants				
Only new-generation antidepressants/others	168	2.1	1.9	ref
Tricyclic antidepressants	72	2.3	1.0	1.03 (0.79, 1.33)
No antidepressants	436	2.4	2.7	1.09 (0.91, 1.31)
Antipsychotics				
Only second-generation antipsychotics	92	2.0	1.0	ref
First-generation antipsychotics	148	2.5	3.5	1.18 (0.92, 1.52)
No antipsychotics	436	2.3	2.1	1.09 (0.87, 1.37)
Other classes (ref = without each drug class)				
Mood stabilizers	117	2.1	0.9	0.91 (0.73, 1.12)
Antiparkinson drugs	71	2.3	1.6	1.01 (0.78, 1.29)
Pain killers	135	2.4	2.4	1.21 (1.00, 1.46)*
Antiallergy drugs	31	3.3	4.3	1.52 (1.12, 2.01)*
Cardiovascular drugs	19	2.2	1.3	0.84 (0.53, 1.27)
Drugs affecting the gut	77	2.0	1.0	0.81 (0.63, 1.03)
Other	120	2.1	1.1	0.91 (0.75, 1.11)
Major diagnosis				
Adjustment disorders	156	2.1	1.4	ref
Bipolar disorders	59	2.2	1.1	1.18 (0.87, 1.58)
Major depressive disorders	174	2.5	2.7	1.18 (0.95, 1.46)
Schizophrenia	72	2.7	2.1	1.35 (1.03, 1.75)*
Other	215	2.3	3.0	1.01 (0.78, 1.30)
Other diagnoses				
Borderline personality disorders	148	2.3	3.4	1.26 (1.00, 1.58)
Other personality disorders	29	2.9	2.4	1.37 (0.97, 1.89)
Anxiety disorders	56	1.9	1.0	0.97 (0.70, 1.33)
Substance use disorders	15	3.5	5.3	1.50 (0.96, 2.24)
Alcohol use disorders	13	2.4	0.8	1.00 (0.58, 1.60)

m = mean; SD = standard deviation; ref = reference group.

* $p < 0.05$.
